# Multiplex real-time PCR using temperature sensitive primer-supplying hydrogel particles and its application for malaria species identification

**DOI:** 10.1371/journal.pone.0190451

**Published:** 2018-01-02

**Authors:** Junsun Kim, Seungwon Jung, Mun Sub Byoun, Changhoon Yoo, Sang Jun Sim, Chae Seung Lim, Sung Woo Kim, Sang Kyung Kim

**Affiliations:** 1 Center for BioMicroSystems, Brain Science Institute, Korea Institute of Science and Technology (KIST), Seoul, Seongbuk-gu, Korea; 2 Department of Chemical and Biological Engineering, Korea University, Seoul, Seongbuk-gu, Korea; 3 Department of Laboratory Medicine, College of Medicine, Korea University, Seoul, Guro-gu, Korea; 4 NanoBioSys Incorporation, Seoul, Geumcheon-gu, Korea; 5 Department of Biomedical Engineering, University of Science and Technology (UST), Daejeon, Yuseong-gu, Korea; University of Helsinki, FINLAND

## Abstract

Real-time PCR, also called quantitative PCR (qPCR), has been powerful analytical tool for detection of nucleic acids since it developed. Not only for biological research but also for diagnostic needs, qPCR technique requires capacity to detect multiple genes in recent years. Solid phase PCR (SP-PCR) where one or two directional primers are immobilized on solid substrates could analyze multiplex genetic targets. However, conventional SP-PCR was subjected to restriction of application for lack of PCR efficiency and quantitative resolution. Here we introduce an advanced qPCR with primer-incorporated network (PIN). One directional primers are immobilized in the porous hydrogel particle by covalent bond and the other direction of primers are temporarily immobilized at so-called 'Supplimers'. Supplimers released the primers to aqueous phase in the hydrogel at the thermal cycling of PCR. It induced the high PCR efficiency over 92% with high reliability. It reduced the formation of primer dimers and improved the selectivity of qPCR thanks to the strategy of 'right primers supplied to right place only'. By conducting a six-plex qPCR of 30 minutes, we analyzed DNA samples originated from malaria patients and successfully identified malaria species in a single reaction.

## Introduction

Real-time PCR has been used for decades as a gold standard for analysing the genes of various diseases.[1–4] The development of multiplex qPCR systems has been necessary for the quantification of various genetic biomarkers in a single trial. However, a conventional solution-phase multiplex qPCR system requires a complex optical system and the number of targets analysed in a single reaction is sometimes deficient for precise diagnosis in samples of limited volume.[5, 6]

Solid phase PCR (SP-PCR) has attracted attention as an alternative to overcome the limitations of conventional qPCR, as its resulting amplicons are confined at the coordinated positions or in suspended particles.[7–12] SP-PCR has potential to increase the number of targets analysed at one time. However, due to its poor reliability and low amplification efficiency, its use is mainly limited to certain applications.[11–15] SP-PCR uses a solid matrix where either or both of the directional primers are immobilized.[9–15] If one primer is immobilized on the surface and the other primer is supplied in the solution, the PCR efficiency is comparable to solution phase PCR.[9, 11] But in this case, the primer supplied from the solution has to be universal among the multiple targets in the multiplex assay.[9, 12, 16] If the primer is not universal, abundant multiple primers might induce cross-talk among the primers and these randomly-produced dimers generally lead to poor reliability of analysis.[17] & **(S1 Fig)** On the other hand, if both primers are immobilized together on the surface (also known as a bridged PCR), the PCR efficiency is dramatically decreased due to the restricted mobility of the immobilized primers **(S2 Fig)**; this results in a configuration that is unsuitable for qPCR, which requires high amplification efficiency and quantitative accuracy.[11]

**Fig 1:**
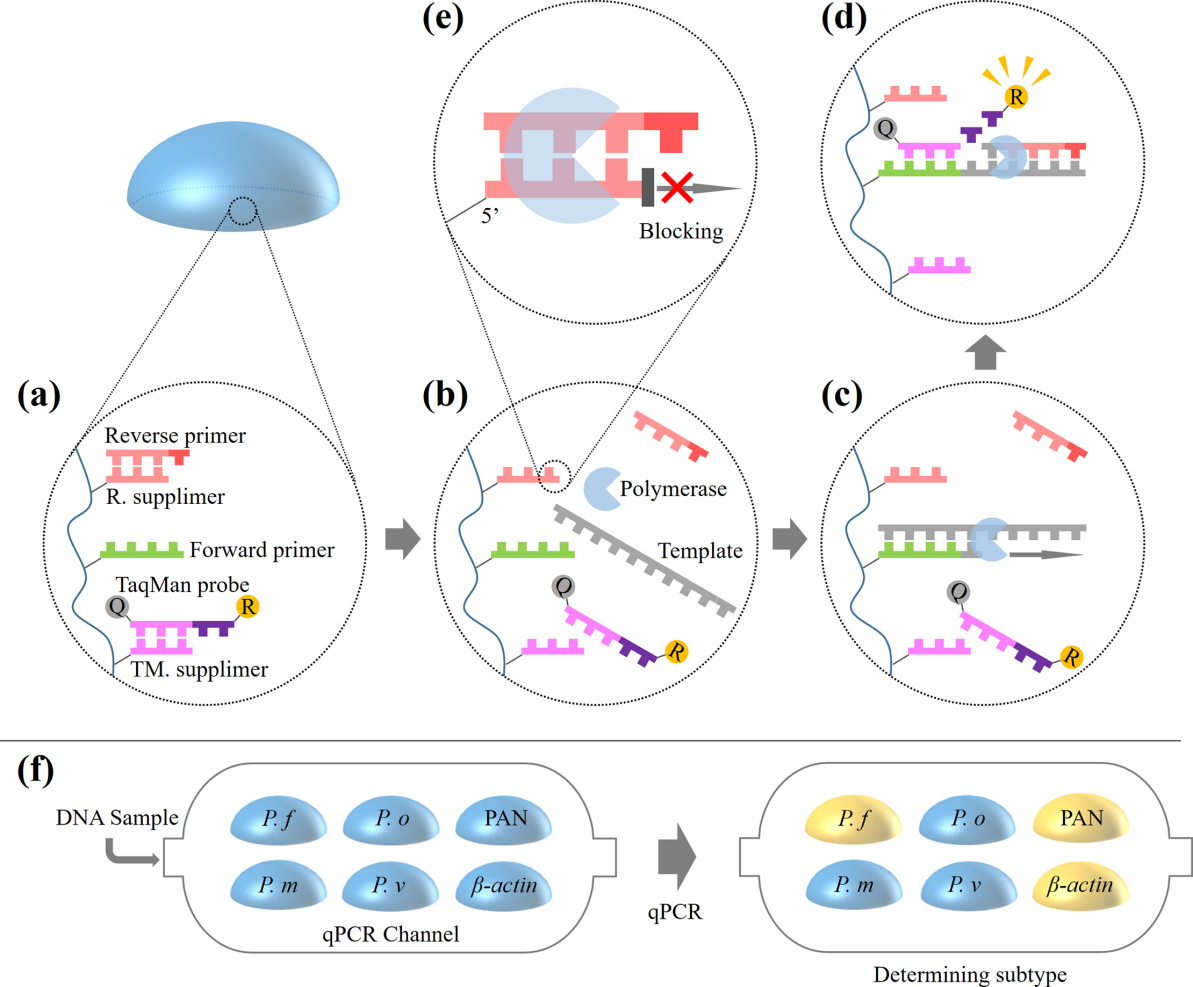
PCR reaction with sPIN particle and multiplex qPCR.

In this paper, we demonstrate a qPCR platform that is appropriate for multiplex DNA analysis with particles supplying target-specific primers and a probe from a temperature-sensitive primer-releasing moiety, which we called a supplimer. A supplimer consists of a complementary sequence of a primer or a probe so it can stably store that specific oligonucleotide by hybridization until the reaction. **(Fig 1A)** In addition, the stored primer and probe can be released from the supplimer at the thermal condition of PCR denature step and participate in the amplification reaction. **(Fig 1B–1D)** This simple but novel method using a supplimer-aided primer-immobilized network (sPIN) shows high PCR efficiency (≥ 92%) which is a prerequisite for accurate quantification. Using this multiplex qPCR system **(Fig 1F)**, practical malaria detection and species identification were successfully demonstrated with DNA samples originating from malaria patients.

## Experimental

### Making of sPIN particle

A pre-polymer solution for the sPIN hydrogel particle was composed of PEG DA (Mn: 700) 20% (v/v), PEG (Mn: 600) 40%, distilled water 35%, and 2-hydroxy-2-methylpropiophenone 5% for a photo-initiator. All the materials mentioned above were purchased from *Sigma-Aldrich*, *USA*.

The acrydite-modified primers and supplimers at the end of 5’ DNA sequence (*IDT*, *IA*, *USA*) were added to the pre-polymer solution to conform to the final concentration of 25 μM. Then, the mixed solution was dropped on the polydimethysiloxane (PDMS) using a liquid handling system (*Arrayer 2000*, *Advanced Technology Inc*., *Korea*) and followed by UV exposure (35 mJ/cm^2^) for cross-linking the PEG DA. The cured sPIN hydrogel particles were fully rinsed with PBS 1X buffer containing 0.05% Tween-20 to remove porogens and unbound primers. Then, all particles were charged with primers (1 μM) and probes (1 μM), which had complementary sequences to specific supplimers, for 5 minutes and the completed sPIN hydrogel particles were stored in a refrigerator at 4°C before use.

### Sample preparation

For *Plasmodium falciparum* 3D7 culture, complete media mixed with RPMI medium 1640 *(Gibco*, *USA)*, 0.5% Albumax I *(Gibco*, *USA)*, 0.5mM hypoxanthine dissolves in 1M NaOH *(Sigma-Aldrich*, *USA)*, 10 ug/ml gentamycin *(Gibco*, *USA)* and 25mM sodium bicarbonate *(Sigma-Aldrich*, *USA)* were used. The *P*. *falciparum* 3D7 strain had obtained from ATCC *(Manassas*, *VA*, *USA)*. To infect the erythrocytes, erythrocytes (45.5 x10^6^ cell/mL) were cultured with the *P*. *falciparum* 3D7 in complete media under a mixed gas phase (O2: CO2: N2 = 5: 5: 90%) at 37°C for 3 weeks. Infection of erythrocytes was evaluated by Giemsa staining and microscopic examination.

Malaria clinical samples (*Plasmodium falciparum and Plasmodium vivax)* were collected from 2014 to 2016 at Korea University Guro Hospital, Republic of Korea. All samples were diagnosed by microscopic examination.

For microscopic examination, thick and thin blood films were prepared using standard protocols. Briefly, after Giemsa staining of blood films, two malaria experts diagnosed the species and density of plasmodial parasites through microscopic examinations. Using both thick and thin blood films, parasitaemia was indirectly calculated using the parasite numbers per 200 WBCs in the blood film, where the WBC counts were determined by the automatic blood cell counter *(Beckman Coulter*, *USA)*.

QIAamp DNA blood mini kit *(Qiagen*, *USA)* was used for extracting gDNA of all sample. Extracting protocol followed by manufacturer’s instructions. Extracted genomic DNA was stored at -80°C.

### qPCR analysis

For the qPCR analysis, a reaction mixture was created containing 1 μl of the DNA sample, 8 μl of the qPCR master mix (*Nanobiosys*, *Korea*), and 7 μl of nuclease free water (*IDT*, *USA*), and is subsequently injected into the qPCR channel, which contains previously injected completed sPIN particles. After 30 minutes of incubation at a constant 4°C, mineral oil was used to isolate each of the PIN hydrogel particles. Finally qPCR was conducted using the *Ultrafast LabChip Real-time PCR G2-4 System*, (*Nanobiosys*, *Korea*) having pre-denature step at 95°C, 10 sec; denature step at 95°C, 4 sec; annealing & extension step at 60°C, 30 sec.

## Results and discussion

### Verifying hybridization ability of supplimer in the sPIN environment

**Fig 2:**
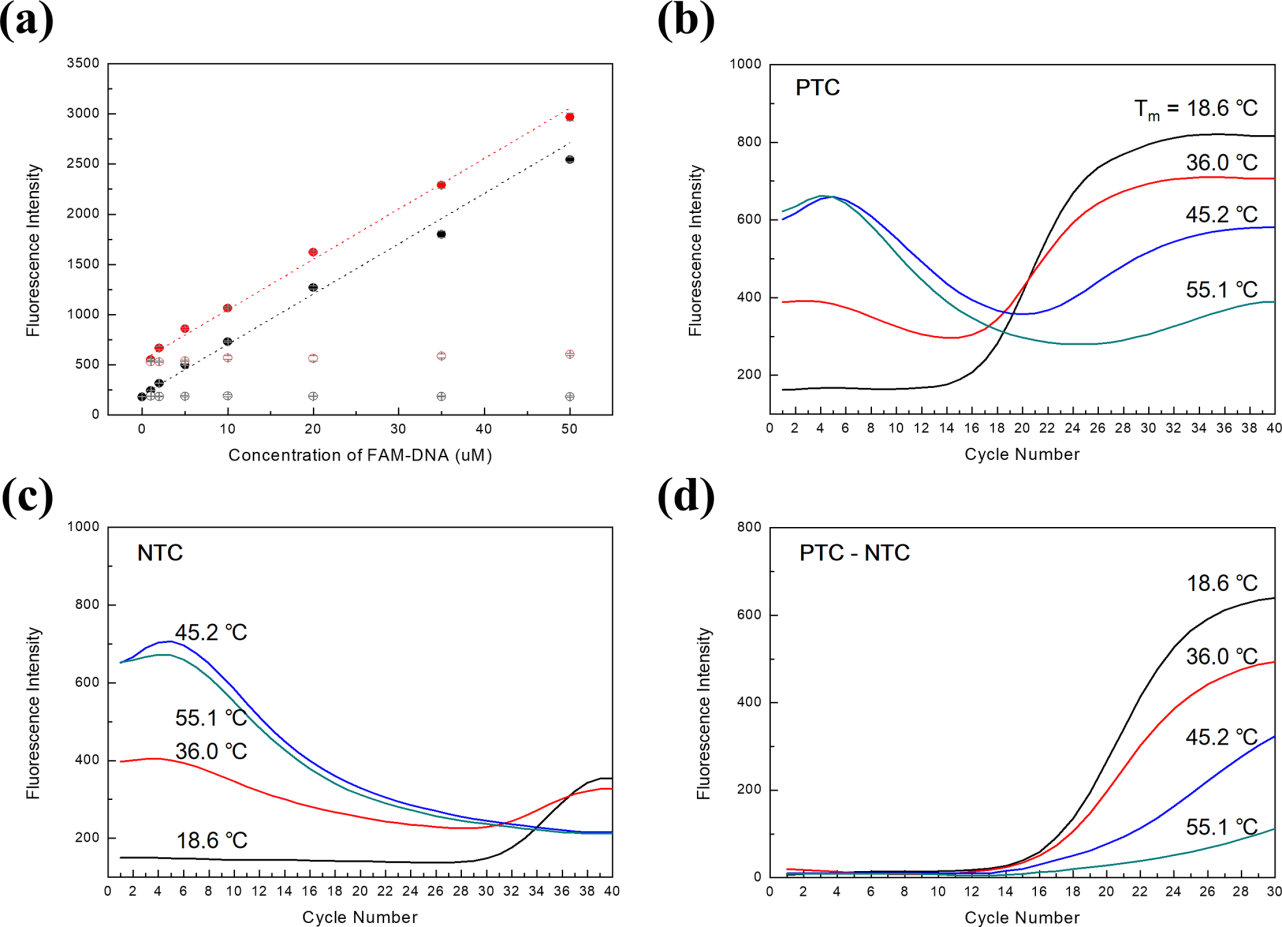
(a) Effective capture of primer on supplimer of sPIN particle. In order to see the capturing efficiency, sPIN particles were prepared with matched (red) or mismatched (black) supplimer to primer. After incubation with FAM-modified primer, both of them showed high intensity depending on the injected FAM-primer concentration (filled red and black circle). After rinsing, mismatched sPIN particle lost the fluorescence (empty black circle), meanwhile matched sPIN particle showed consistently remained fluorescence (empty red circle). It was caused by the stable binding between the primer and corresponding supplimer. (b) Positive qPCR graph of each sPIN particle using supplimers with different T_m_. (c) Negative qPCR graph of each sPIN particle with supplimers of different T_m_. (d) Fluorescent signal subtracted No Template Control (NTC) signal from Positive Template Control (PTC) showing regular S-shaped curves.

To verify hybridization ability and specificity of supplimer, three kinds of PIN particles were prepared; 1) sPIN particles having supplimers which were matched-sequence with primer (25 μM, 5’/acryd/-AGTACTCCGT-/phos/3’), 2) sPIN particles with mismatched-sequence of supplimers (25 μM, 5’/acryd/-ACCCATCAAG-/phos/3’), 3) PIN particles without supplimers. The particles were immersed respectively in aqueous solutions with varied concentrations of FAM-modified primers (5’-FAM-GCCGATCCACACGGAGTACT-3’) to be loaded with the designated reverse primers under active agitation. The fluorescent intensities of the particles were measured before and after the rinsing step with a PBS buffer. The differential intensity is believed to represent the pure primers specifically captured by the supplimers. Before rinsing, matched sPIN particles showed a higher fluorescent intensity than the others. This is caused by the additional accumulation of complimentary primers through the specific hybridization in the particle.[18] After a stringent rinsing step, the mismatched- or no-supplimer particles hardly showed fluorescence because the FAM-modified primers were mostly removed by rinsing step. The matched particles, however, still remained fluorescent after rinsing, indicating the stable binding between the primer and supplimer. **(Fig 2A).**

This result indicates that the primer-supplimer binding is sequence-specific and endures active agitation. Interestingly, the difference between matched and mismatched particles in fluorescence intensity at each concentration of FAM-DNA was kept constant. The final intensities after rinsing were also uniform regardless of the concentration of FAM-DNA, provided the concentration exceeds 1 μM. This uniformity is attributed to the saturation of the supplimers in the particle with primers, i.e., the amount of supplimer is the limiting factor of the resulting amount of hybridized primers.

### Comparing hybridization stability and PCR aspects by varying melting temperature of supplimer

Hybridized primers at supplimers must meet two conditions. First, they should be tightly hybridized until PCR is started. Lose binding between primer and supplimer leads to the loss of primers and then it will aggravate reliability of assay. Second, the hybridized primers should be released effectively from the very beginning of PCR. A high T_m_ of the supplimer might hinder annealing between the primers and the templates due to the possible re-hybridization of primers to supplimers. For these two conditions, therefore, the T_m_ of the supplimer should be optimized to evade competition during the annealing step while maintaining the storage stability of primers at room temperature.

Since the supplimer is based on DNA hybridization, the binding affinity of a primer-supplimer complex can be modulated by varying the length of the complimentary supplimer. Four different supplimers were compared, which was denatured at varying temperatures (T_m_ = 18.6, 36.0, 45.2, and 55.1°C, the sequences are described in the **S3 Fig**), through the hybridization experiment with FAM-modified complementary DNA at room temperature. The supplimers with a T_m_ of 36.0, 45.2, and 55.1°C showed no significant difference in fluorescence intensities after rinsing, but the fluorescence of the sPIN particle with a supplimer of 18.6°C in T_m_ clearly decreased compared with the others, which means that the storage of that primer is unstable under room temperature conditions. On the basis of this result, we can conclude that the supplimers over 30°C in T_m_ are favored for the hybridization of the primer at room temperature. A further study on the long-term storage stability of the supplimer-primer complex supported this result, not showing any deteriorate performances after one month stored at 4°C. **(S4 Fig)**

To evaluate PCR aspects according to different melting temperature of supplimer, four different T_m_ of supplimers (T_m_ = 18.6, 36.0, 45.2, and 55.1°C) were used to make respective sPIN particles. At the same time, same concentration of forward primers (5’/acryd/-CCTGGCACCCAGCACAAT-3’, 25 μM) were also immobilized to each sPIN particles by covalently bond. After that, reverse primers (5’-GCCGATCCACACGGAGTACT-3’, 1 μM) were fully hybridized to each particle and the unbound primers were rinsed off with PBS buffer. The SYBR green I PCR master mix with template was injected into the particle-preloaded channels. After a 30-minute incubation, individual particles were isolated simply by injecting mineral oil.

As in the usual qPCR process, fluorescence was observed in every thermal cycle. **(Fig 2B and 2C)** Contrarily, the initial fluorescence intensity was higher than the one from bare PIN particles and it decreased for the initial 10 cycles. After that, the intensity increased, as in general amplification curves. In case of NTC, the initial fluorescence gradually dropped to the final cycle. This initial fluorescence was ascribed to the supplimer-primer complex. Since the intercalating dye such as SYBR green I is activated and emits strong fluorescence through binding to the structure of double helix, it becomes fluorescent in the primer-supplimer complex as well. Accordingly, as the binding sequences between supplimer and primer are longer which have higher T_m_, the primer-supplimer complex having more intercalating sites of the SYBR green I takes on stronger background fluorescence. According to **Fig 2C**, initial intensity became higher as the T_m_ of the supplimer increased. The gradual decrease of fluorescence illustrates the reduction of the primer-supplimer complex with the increasing number of thermal cycles. Therefore, the fluorescence signal of the PTC appearing in **Fig 2B** is the summation of signals from the amplification of the target templates and from the remaining primer-supplimer complex. In order to differentiate the fluorescence only from amplification, we subtracted the NTC signal from the PTC signal. As a result, the usual amplification graphs were obtained, consisting of the three phases: the exponential, linear, and a plateau phases. **(Fig 2D)** This means that the amplification in a sPIN particle is similar to a usual qPCR. Interestingly, a higher T_m_ of supplimer showed increased Ct value in parallel with the lower final intensity. It is likely that supplimers of higher T_m_ compete with templates over primers and interrupted the effective amplification of the templates. In considering both the storage stability and amplification efficiency, we concluded that the optimal T_m_ of the supplimer resides in the 30 to 35°C range.

### Calculating PCR efficiency and measuring limit of detection

Initial fluorescence from primer-supplimer complex can be avoided with TaqMan probe assays. The TaqMan probe is known to provide better specificity than the SYBR green I assay.[19, 20] We also examined both assays by comparing qPCR data with a low concentration of human gDNA sample and confirmed that the TaqMan probe assay is more specific and reliable than the SYBR green I assay. **(S5 Fig)** Especially, the TaqMan assay proves its value when complex nucleic acids comprising human DNA samples are analysed.

**Fig 3:**
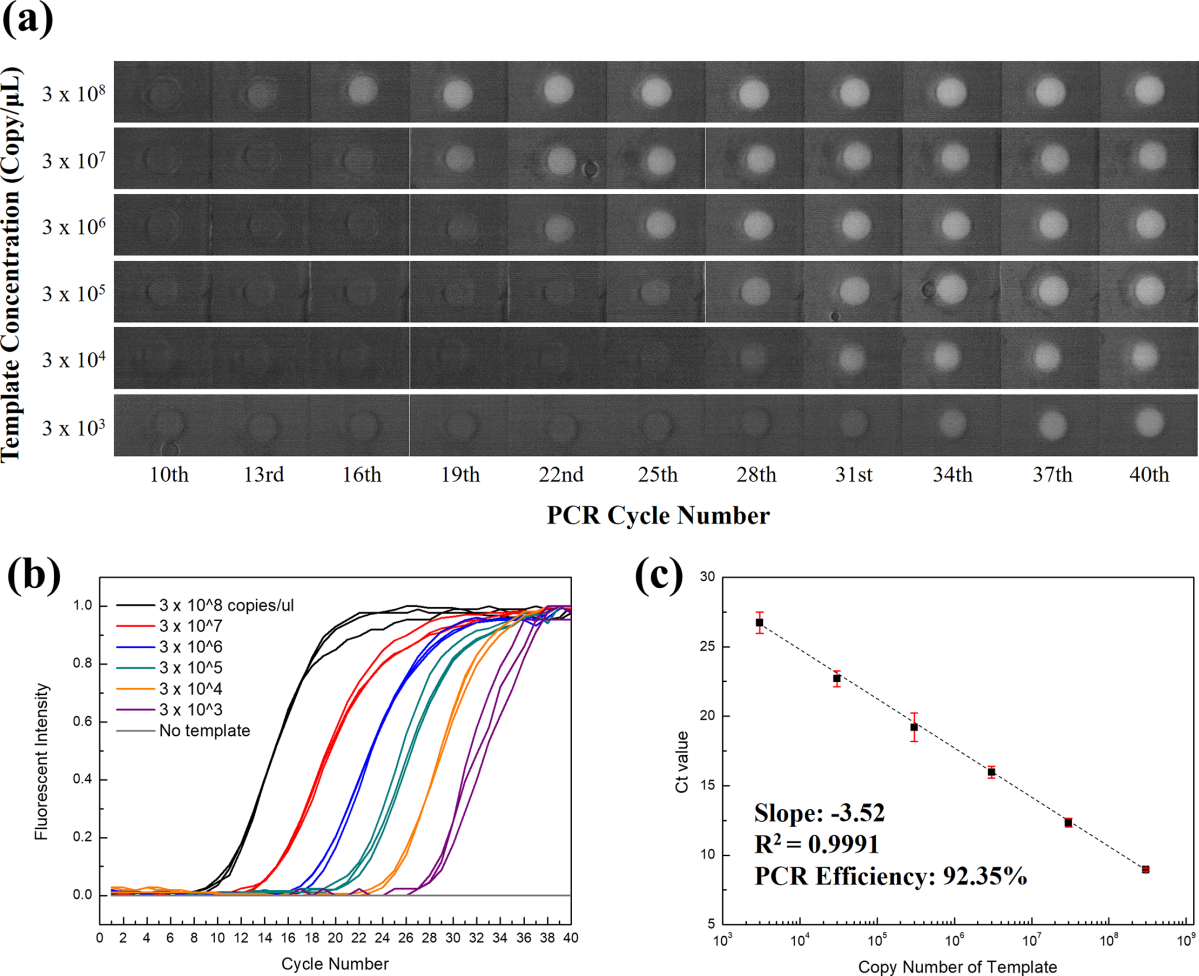
Fluorescent snapshots of sPIN particle at each cycle of qPCR (a) and its graphs (b) according to serial dilution of template. In particular, the graphs consist of triplicate sets for each concentration. (c) Calibration curve showing the Ct values of sPIN qPCR with serial dilution. This shows a constant interval of 3.5 for the Ct value, indicating that sPIN qPCR is stable and reliable.

In the same manner as an R supplimer, 25 μM of TaqMan supplimer (TM supplimer) was also immobilized when the sPIN particle was made, i.e., the forward primer, R supplimer and TM supplimer were immobilized in the sPIN particle. Then, the reverse primer (1 μM) and TaqMan probe (1 μM) were captured to each supplimer through the incubation. As a result of serial dilution, sPIN was able to analyse concentrations of the template as low as 3*10^3^ copies/μl, showing high a quantification stability, with a deviation of less than 5% in Ct value from the triplicated results. **(S6 Fig)** Its PCR efficiency was calculated to be 92.35%, according to the linear calibration curve, which was comparable to conventional solution based qPCR method. **(Fig 3)** & **(S7 Fig).**

Interestingly, template concentrations under 1*10^3^ copies/μl resulted in some of the sPIN particles with “off” signal. When 1*10^3^ copies of a target template are injected in the PCR channel and uniformly distributed through, a single sPIN of 20 nl volume contains 1 copy of the template on average. Practically, almost half of sPIN particles are occupied by a couple of templates and others are unoccupied. Through the conventional solution qPCR of 20 μl of reaction volume, 3*10^2^ copies/μl of the template could be analysed under the same experimental condition. The limit of quantification in sPIN can be improved with the number of sPIN particles in a single assay. This is similar to the on/off signal from general digital PCRs.

### Multiplex qPCR for malaria species identification

**Fig 4:**
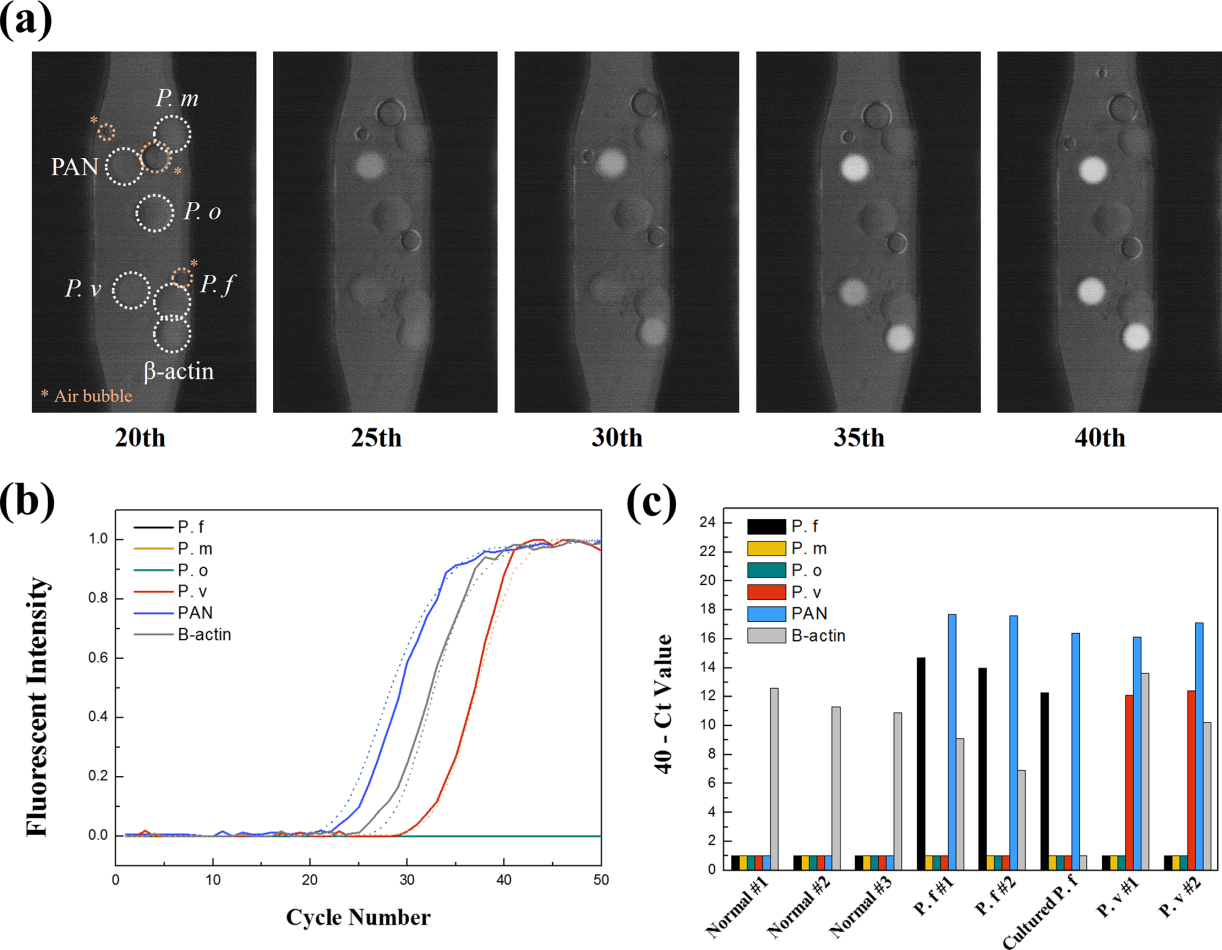
(a) Fluorescent image sequence of multiplex qPCR with a synthetic template. Six different sPIN particles (*P*. *f*, *P*. *m*, *P*. *o*, *P*. *v*, PAN, and β-actin) were located in single channel. During qPCR, sPIN particles targeting PAN, *P*. *v*, and β-actin selectively showed fluorescent signals. Although there were air bubbles in the channel which were inevitable when the plastic chip was used, they did not interfere fluorescent signal of sPIN particles. That’s because the fluorescent signal was measured in the sPIN particles only. (b) qPCR graph analyzed with DNA sample obtained from *P*. *v* patients. Solid lines are results of multiplex assay and dotted lines are for the single-plex assay. They showed no significant difference on amplification curves and no other cross-talk. (c) The result results of eight different clinical tests. The Y-axis was set to ‘40-Ct value’ to emphasize that the high height of a column corresponds to a high concentration of the target. A value of one on the y-axis means that there was no signal at all for 50 PCR cycles.

The use of several sPIN particles in a channel permits the multiplex qPCR in a single reaction. In this paper, malaria was targeted for multiplex qPCR, which has four typical subtypes; *Plasmodium falciparum* (*P*. *f)*, *Plasmodium malariae (P*. *m)*, *Plasmodium ovale (P*. *o)* and *Plasmodium vivax (P*. *v*).[2, 4, 21–23] Since different drugs should be prescribed according to malaria subtype, rapid identification is important for effective treatment of patients.[24, 25] In this assay, the cocktail of the particles in a channel determined not only whether or not the patient is infected by malaria, but also identify its species at the same time. Six kinds of sPIN particles comprised one multiplex qPCR set: PAN for malaria positive control, four different subtypes, and β-actin for human positive control.[25, 26] Before the introduction of a clinical sample, a mixture of synthetic templates was analysed to verify the performance, as shown in **Fig 4A**. Each particle brightened in response to the presence of target templates and their Ct values conformed to the intended concentration of the synthetic templates.

Moving towards a real world application, eight different clinical samples were analysed using this multiplex assay. Each multiplex qPCR was conducted with a small sample of 1 μl and it took less than 25 minutes for 40 PCR cycles. **Fig 4B** shows the graphs of a multiplex assay of DNA extracted from a malaria patient (subtype = *P*. *v*). The specificity of the sPIN qPCR was evidenced by overlapped graphs between the multiplex (solid line) and singleplex (dotted line) assays. This means there was no cross-talk between the genetic targets. Seven more samples were tested, six originating from human blood and one from a malaria culture (subtype = *P*. *f*). For a total of 8 samples, we could discriminate the infectious status of patients and their species, as shown in **Fig 4C**. In the three normal (uninfected) samples, only β-actin particles made fluorescence signals. The three other samples showed positive signals from particles for *P*. *f* and PAN, as well as β-actin, determining both the malaria infection and the subtype of *P*. *f*. In the case of two samples from *P*. *v* patients, the signals for *P*. *v* instead of *P*. *f* appeared. Even though no multiplex qPCR was conducted with the sample of *P*. *m*, *P*. *o* and mixed species infection cases, we could anticipate each corresponding sPIN particles would be reacted, as considering that *P*. *f* and *P*. *v* infection was successfully identified.

## Conclusion

We demonstrate a new strategy for overcoming the limitations of conventional SP-PCR by enhancing its efficiency and reliability. In order to compensate for the low mobility of the immobilized primer, one primer was reversibly bound to the supplimer, consisting of a complementary sequence of the primer and released in time to participate in the reaction during PCR. A TaqMan probe qPCR with sPIN particles showed a high efficiency (92.35%) of amplification reaction and excellent reliability with a small variation of less than 5%. Multiplex sPIN qPCR was carried out by loading the different particles in a single channel, and successful identification of malaria species was demonstrated with clinical samples. This multiplex sPIN qPCR is not limited to malaria but generally applicable to other targets through simple constitution of the supplimers matched with their primer and probe. Therefore, this flexibility will have our method be used for analyzing other nucleic acid targets in many purposes.

## Supporting information

S1 Fig Multiplex qPCR with mixed primers. This result shows the problem in multiplex qPCR when the primers are supplied as mixed. The reverse primers for each target were mixed and supplied to channel where each forward primer immobilized particle was located. Different from sPIN qPCR showing no signal with no template, false positive signals were generated in mixed case at severe level even though qPCR was conducted without any template. These false positive signals were made by the formation of dimer between primers and it means that each different reverse primer should be independently supplied into corresponding particle using supplimer.
(DOCX)

S2 Fig Comparison of three different types of PIN qPCR. When both forward and reverse primers were immobilized to solid matrix (Black line), qPCR signal was not detected because PCR efficiency was extremely low. This is because restricted mobility of the immobilized primers could not engage enzymatic reaction favorably. On the other hand, when forward primer was immobilized to solid matrix and reverse primer was supplied in solution phase (Red line), the graph showed S-shape signal, meaning high PCR efficiency thanks to recovered mobility of reverse primer by releasing from supplimer. sPIN particle (Blue line) showed almost same performance as com-pared to single primer immobilized particle. Therefore, we can conclude that the use of supplimer is as efficient as supplement from solution.
(DOCX)

S3 Fig Sequence of primer and supplimer. (DOCX)

S4 Fig Storage stability test of supplimer-primer complex in sPIN. A ‘Ct value variation standard level’ was calculated to assess whether the qPCR performance of sPIN is changed, from 10 times of independent qPCR data analyzing same concentration of target template. We set the Ct value variation standard level to ±0.5.
Storage stability test of sPIN particle was conducted until 30 days after it’s made. R supplimer, which has 36.0°C of melting temperature was used to make sPIN particle. The sPIN particles were stored at 4°C after hybridization of R primer. Considering a Ct value variation level of storage stability test was ±0.15 which was under the standard level, we concluded that there were not meaningful changes of Ct value until 30 days after.
(DOCX)

S5 Fig Comparison of SYBR green I and TaqMan based assay using human gDNA sample. We compared both SYBR green I and TaqMan based assay to examine specificity and reliability. Human gDNA sample was used and TaqMan probe assay showed more consistent Ct value (Standard deviation: ±0.4) than SYBR green I assay (Standard deviation: ±1.8). Therefore, we concluded that TaqMan probe assay is more suitable than SYBR green I assay for clinical applications with more quantitative stability.
(DOCX)

S6 Fig Repeatability test. qPCR with ten sPIN particles were independently conducted with same concentration of template. As a result, it showed highly producible performance having standard deviation ±0.5 in Ct value.
(DOCX)

S7 Fig Sensitivity comparison between sPIN particle and conventional solution phase qPCR. Since PIN particle contains only 20 nL of reaction volume, it is too limited to use all of template in PCR cocktail solution which is generally 10~20 μL. For that reason, limit of detection level of sPIN particle looks poorer than conventional solution phase qPCR. However, if considering template copies per each single PIN particle, its PCR performance is excellent. Even several copies were enough to conduct PCR reaction in PIN particle. When template concentration is lowered than 3*10^3^ copies/μL to be below one copy per PIN particle, it showed on/off signal among the particles, in other words, digital signal.
(DOCX)
