# Supplementary material for: Multiplex real-time PCR using temperature sensitive primer-supplying hydrogel particles and its application for malaria species identification
Source: PLoS One. 2018 Jan 2;13(1):e0190451. doi: 10.1371/journal.pone.0190451 (PMC5749795; doi:10.1371/journal.pone.0190451)
Supplement: S1 Fig — This result shows the problem in multiplex qPCR when the primers are supplied as mixed. The reverse primers for each target were mixed and supplied to channel where each forward primer immobilized particle was located. Different from sPIN qPCR showing no signal with no template, false positive signals were generated in mixed case at severe level even though qPCR was conducted without any template. These false positive signals were made by the formation of dimer between primers and it means that each different reverse primer should be independently supplied into corresponding particle using supplimer. (DOCX) [file pone.0190451.s001.docx]

**S1 Fig. Multiplex qPCR with mixed primers**


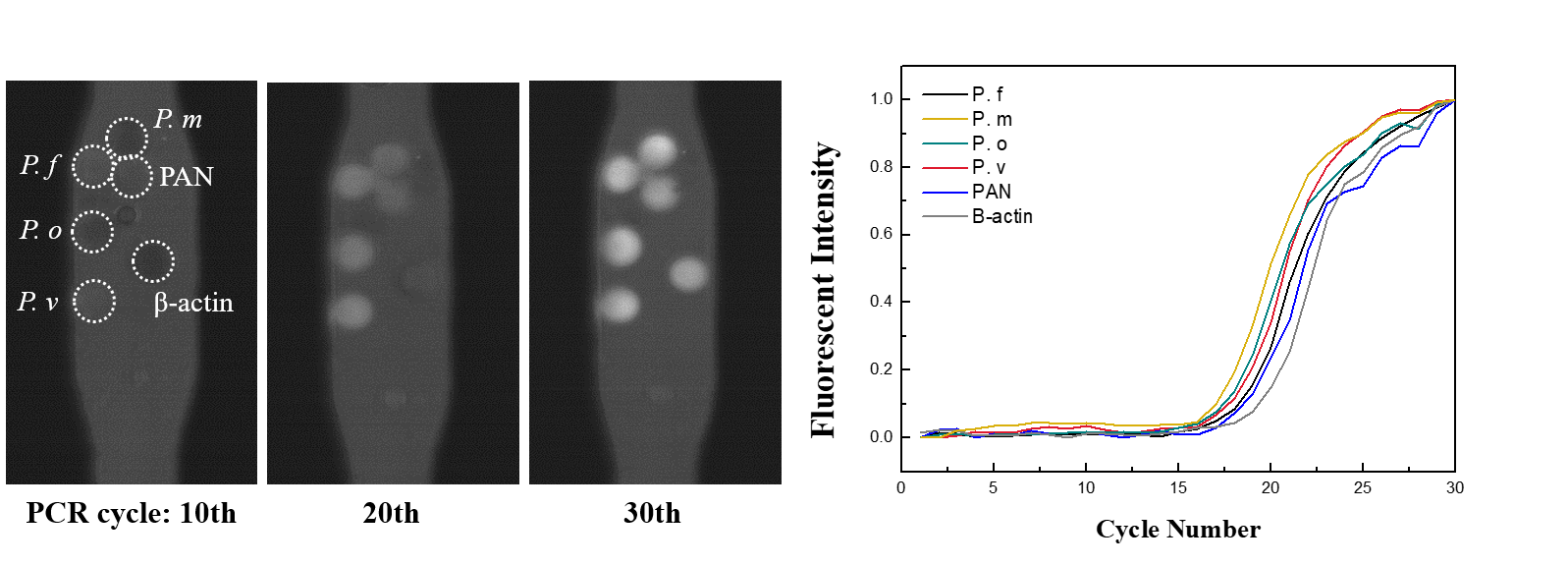


This result shows the problem in multiplex qPCR when the primers are supplied as mixed. The reverse primers for each target were mixed and supplied to channel where each forward primer immobilized particle was located. Different from sPIN qPCR showing no signal with no template, false positive signals were generated in mixed case at severe level even though qPCR was conducted without any template. These false positive signals were made by the formation of dimer between primers and it means that each different reverse primer should be independently supplied into corresponding particle using supplimer.
