# Supplementary material for: Multiplex real-time PCR using temperature sensitive primer-supplying hydrogel particles and its application for malaria species identification
Source: PLoS One. 2018 Jan 2;13(1):e0190451. doi: 10.1371/journal.pone.0190451 (PMC5749795; doi:10.1371/journal.pone.0190451)
Supplement: S2 Fig — When both forward and reverse primers were immobilized to solid matrix (Black line), qPCR signal was not detected because PCR efficiency was extremely low. This is because restricted mobility of the immobilized primers could not engage enzymatic reaction favorably. On the other hand, when forward primer was immobilized to solid matrix and reverse primer was supplied in solution phase (Red line), the graph showed S-shape signal, meaning high PCR efficiency thanks to recovered mobility of reverse primer by releasing from supplimer. sPIN particle (Blue line) showed almost same performance as com-pared to single primer immobilized particle. Therefore, we can conclude that the use of supplimer is as efficient as supplement from solution. (DOCX) [file pone.0190451.s002.docx]

**S2 Fig. Comparison of three different types of PIN qPCR**

When both forward and reverse primers were immobilized to solid matrix (Black line), qPCR signal was not detected because PCR efficiency was extremely low. This is because restricted mobility of the immobilized primers could not engage enzymatic reaction favorably. On the other hand, when forward primer was immobilized to solid matrix and reverse primer was supplied in solution phase (Red line), the graph showed S-shape signal, meaning high PCR efficiency thanks to recovered mobility of reverse primer by releasing from supplimer. sPIN particle (Blue line) showed almost same performance as com-pared to single primer immobilized particle. Therefore, we can conclude that the use of supplimer is as efficient as supplement from solution.
