# Supplementary material for: Multiplex real-time PCR using temperature sensitive primer-supplying hydrogel particles and its application for malaria species identification
Source: PLoS One. 2018 Jan 2;13(1):e0190451. doi: 10.1371/journal.pone.0190451 (PMC5749795; doi:10.1371/journal.pone.0190451)
Supplement: S3 Fig — (DOCX) [file pone.0190451.s003.docx]

**S3 Fig. Sequence of primer and supplimer**

Forward primer: 5’/acryd/-CCTGGCACCCAGCACAAT

Reverse primer: 5’-GCCGATCCACACGGAGTACT-3’

R supplimer (Tm=18.6 °C): 5’/acryd/-AGTACTCC-/phos/3’

R supplimer (Tm=36.0 °C): 5’/acryd/-AGTACTCCGT-/phos/3’

R supplimer (Tm=45.2 °C): 5’/acryd/-AGTACTCCGTGT-/phos/3’

R supplimer (Tm=55.1 °C): 5’/acryd/-AGTACTCCGTGTGGA-/phos/3’
