# Supplementary material for: Multiplex real-time PCR using temperature sensitive primer-supplying hydrogel particles and its application for malaria species identification
Source: PLoS One. 2018 Jan 2;13(1):e0190451. doi: 10.1371/journal.pone.0190451 (PMC5749795; doi:10.1371/journal.pone.0190451)
Supplement: S4 Fig — A ‘Ct value variation standard level’ was calculated to assess whether the qPCR performance of sPIN is changed, from 10 times of independent qPCR data analyzing same concentration of target template. We set the Ct value variation standard level to ±0.5. Storage stability test of sPIN particle was conducted until 30 days after it’s made. R supplimer, which has 36.0°C of melting temperature was used to make sPIN particle. The sPIN particles were stored at 4°C after hybridization of R primer. Considering a Ct value variation level of storage stability test was ±0.15 which was under the standard level, we concluded that there were not meaningful changes of Ct value until 30 days after. (DOCX) [file pone.0190451.s004.docx]

**S4 Fig. Storage stability test of supplimer-primer complex in sPIN**

A ‘Ct value variation standard level’ was calculated to assess whether the qPCR performance of sPIN is changed, from 10 times of independent qPCR data analyzing same concentration of target template. We set the Ct value variation standard level to ±0.5.

Storage stability test of sPIN particle was conducted until 30 days after it’s made. R supplimer, which has 36.0 °C of melting temperature was used to make sPIN particle. The sPIN particles were stored at 4 °C after hybridization of R primer. Considering a Ct value variation level of storage stability test was ±0.15 which was under the standard level, we concluded that there were not meaningful changes of Ct value until 30 days after.
