# Supplementary material for: Multiplex real-time PCR using temperature sensitive primer-supplying hydrogel particles and its application for malaria species identification
Source: PLoS One. 2018 Jan 2;13(1):e0190451. doi: 10.1371/journal.pone.0190451 (PMC5749795; doi:10.1371/journal.pone.0190451)
Supplement: S5 Fig — We compared both SYBR green I and TaqMan based assay to examine specificity and reliability. Human gDNA sample was used and TaqMan probe assay showed more consistent Ct value (Standard deviation: ±0.4) than SYBR green I assay (Standard deviation: ±1.8). Therefore, we concluded that TaqMan probe assay is more suitable than SYBR green I assay for clinical applications with more quantitative stability. (DOCX) [file pone.0190451.s005.docx]

**S5 Fig. Comparison of SYBR green I and TaqMan based assay using human gDNA sample**

We compared both SYBR green I and TaqMan based assay to examine specificity and reliability. Human gDNA sample was used and TaqMan probe assay showed more consistent Ct value (Standard deviation: ±0.4) than SYBR green I assay (Standard deviation: ±1.8). Therefore, we concluded that TaqMan probe assay is more suitable than SYBR green I assay for clinical applications with more quantitative stability.
