# Supplementary material for: Multiplex real-time PCR using temperature sensitive primer-supplying hydrogel particles and its application for malaria species identification
Source: PLoS One. 2018 Jan 2;13(1):e0190451. doi: 10.1371/journal.pone.0190451 (PMC5749795; doi:10.1371/journal.pone.0190451)
Supplement: S6 Fig — qPCR with ten sPIN particles were independently conducted with same concentration of template. As a result, it showed highly producible performance having standard deviation ±0.5 in Ct value. (DOCX) [file pone.0190451.s006.docx]

**S6 Fig. Repeatability test**

qPCR with ten sPIN particles were independently conducted with same concentration of template. As a result, it showed highly producible performance having standard deviation ±0.5 in Ct value.
