# Supplementary material for: Multiplex real-time PCR using temperature sensitive primer-supplying hydrogel particles and its application for malaria species identification
Source: PLoS One. 2018 Jan 2;13(1):e0190451. doi: 10.1371/journal.pone.0190451 (PMC5749795; doi:10.1371/journal.pone.0190451)
Supplement: S7 Fig — Since PIN particle contains only 20 nL of reaction volume, it is too limited to use all of template in PCR cocktail solution which is generally 10~20 μL. For that reason, limit of detection level of sPIN particle looks poorer than conventional solution phase qPCR. However, if considering template copies per each single PIN particle, its PCR performance is excellent. Even several copies were enough to conduct PCR reaction in PIN particle. When template concentration is lowered than 3*103 copies/μL to be below one copy per PIN particle, it showed on/off signal among the particles, in other words, digital signal. (DOCX) [file pone.0190451.s007.docx]

**S7 Fig. Sensitivity comparison between sPIN particle and conventional solution phase qPCR**


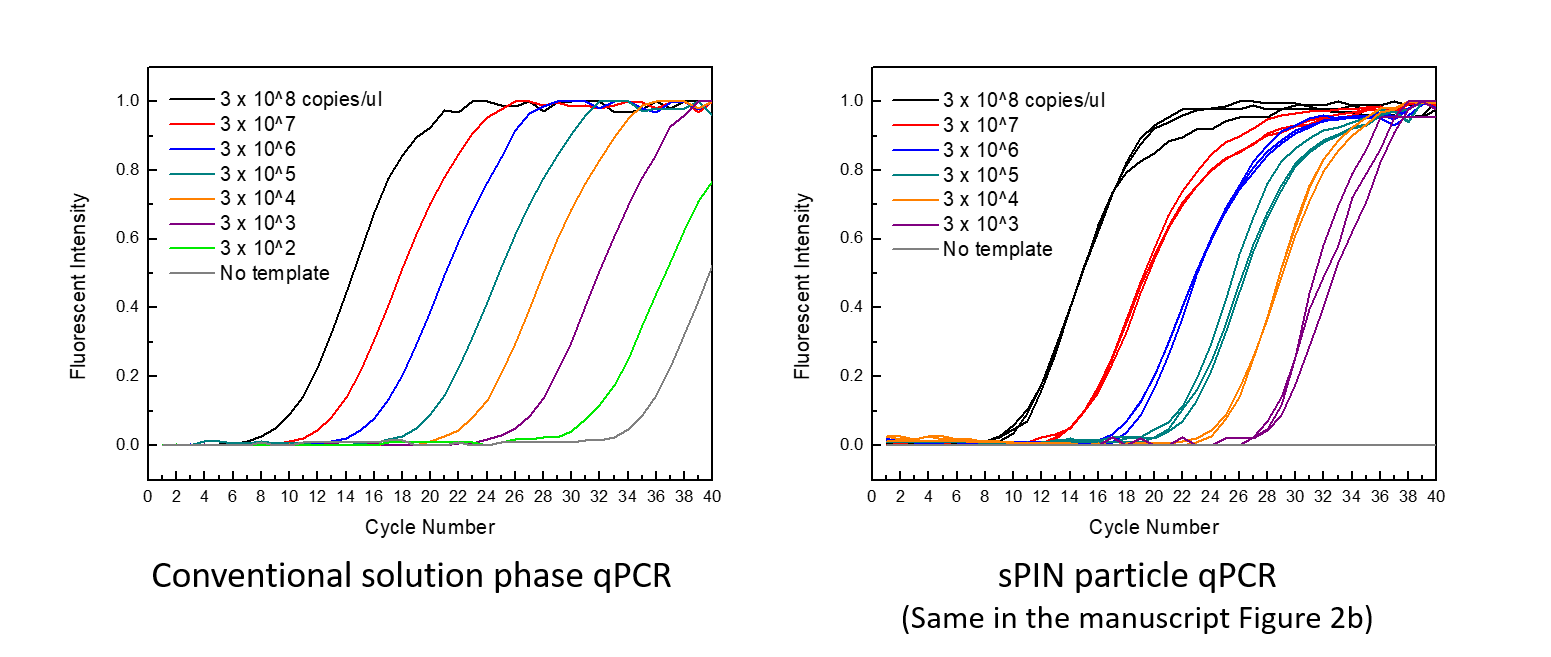


Since PIN particle contains only 20 nL of reaction volume, it is too limited to use all of template in PCR cocktail solution which is generally 10~20 μL. For that reason, limit of detection level of sPIN particle looks poorer than conventional solution phase qPCR. However, if considering template copies per each single PIN particle, its PCR performance is excellent. Even several copies were enough to conduct PCR reaction in PIN particle. When template concentration is lowered than 3*10^3^ copies/μL to be below one copy per PIN particle, it showed on/off signal among the particles, in other words, digital signal.
